# P-13. Clinical Outcomes and Predictors of Mortality in AmpC-producing Gram-negative Bacteremia at a Tertiary Academic Medical Center

**DOI:** 10.1093/ofid/ofaf695.244

**Published:** 2026-01-11

**Authors:** David S Burgess, Katie B Olney, Donna R Burgess

**Affiliations:** University of Kentucky, Lexington, KY; University of Kentucky HealthCare, Lexington, KY; UK HealthCare, Lexington, KY

## Abstract

**Background:**

Infections due to AmpC-producing Gram-negative organisms present therapeutic challenges and are associated with significant morbidity and mortality. We aimed to characterize the clinical features, outcomes, and predictors of mortality in patients with bacteremia caused by organisms at high risk for AmpC induction at a large academic medical center.

**Methods:**

This retrospective cohort study included adults with Gram-negative bloodstream infections from July 2022 through December 2024. Patients with bacteremia caused by *K. aerogenes*, *E. cloacae*, or *C. freundii* were included. Clinical data, microbiology results, comorbidities, treatment characteristics, and outcomes were collected. A stepwise multiple linear regression model identified independent predictors of mortality.

**Results:**

Among 970 patients with Gram-negative bacteremia, 81 (8.4%) were due to *K. aerogenes*, *E. cloacae*, or *C. freundii*. Median (IQR) age was 56 yrs (42, 68), and 81.5% of patients were White. Most cases (50.6%) were hospital-acquired, and ICU admission occurred in 53.1%. The in-hospital mortality rate was 13.6%, and 90-day readmission rate was 54.3%. Median hospital and ICU length of stay was 17 days (9, 30.5) and 8.5 days (4.1, 17.6), respectivley. ID consultation occurred in 55.6% of cases, with a median time to consult of 25.3 hrs (0.7, 73.1). Median duration of antibiotic therapy was 8.5 days (6.8, 12.9). Median time to ePlex® rapid diagnostic was 15.5 hrs (13.0, 20.1), and time to susceptibility results was 3.8 days (2.7, 5.8). Common comorbidities included COPD (39.5%), CHF (33.3%), CVD (27.2%), diabetes (25.9%), and liver disease (18.5%). In regression modeling, higher SOFA score (p = 0.006), longer time to susceptibility results (p = 0.011), and prolonged ICU stay (p = 0.034) were independently associated with increased mortality.

**Conclusion:**

AmpC-producing Gram-negative bacteremia was associated with high ICU use, prolonged hospitalization, and notable mortality. SOFA score, delayed susceptibility data, and ICU stay were independently associated with mortality. These findings underscore the importance of early recognition, rapid diagnostics, and timely antimicrobial optimization to improve outcomes.

**Disclosures:**

All Authors: No reported disclosures

